# Fidelity in RNA-based recognition of transposable elements

**DOI:** 10.1098/rstb.2018.0168

**Published:** 2018-11-05

**Authors:** Ilaria Ugolini, Mario Halic

**Affiliations:** Department of Biochemistry and Gene Center, LMU Munich, 81377 Munich, Germany

**Keywords:** *S. pombe*, PARN, transposon, heterochromatin, piRNA, RNAi

## Abstract

Genomes are under constant threat of invasion by transposable elements and other genomic parasites. How can host genomes recognize these elements and target them for degradation? This requires a system that is highly adaptable, and at the same time highly specific. Current data suggest that perturbation of transcription patterns by transposon insertions could be detected by the RNAi surveillance pathway. Multiple transposon insertions might generate sufficient amounts of primal small RNAs to initiate generation of secondary small RNAs and silencing. At the same time primal small RNAs need to be constantly degraded to reduce the level of noise small RNAs below the threshold required for initiation of silencing. Failure in RNA degradation results in loss of fidelity of small RNA pathways and silencing of ectopic targets.

This article is part of the theme issue ‘5′ and 3′ modifications controlling RNA degradation’.

## 1. Recognition of transposable elements

Genomes are under constant threat of invasion by transposable elements and other genomic parasites. These foreign genomic elements will use the host machinery for their own expression and proliferation. Their transposition can lead to disruption of endogenous genes and regulatory elements. On the other hand, proliferation of transposable elements can cause mutations that might be beneficial for the host in stress conditions and might increase its survival. In this regard, repetitive and transposable elements are one of the major drivers of genome evolution and diversity [1–3]. In many cases, transposons are adopted by the genome and are used as regulatory elements for gene expression and RNA processing [1,4–7].

To protect themselves from proliferation of transposable sequences, genomes have evolved elaborate mechanisms that silence their expression, which is essential for genome stability and cell growth. It is particularly important to protect the genetic information that will be passed to the next generation. Consequently, genome protection pathways are more sophisticated in single cell organisms and in germline cells of multicellular organisms. How can genomes recognize transposable elements and silence them is a fundamental unanswered question. This is not trivial for the host since transposons are very diverse, limiting strategies that recognize a specific transposon sequence. Transposons also use different mechanisms for their proliferation, restricting their identification based on recognition of enzymatic reactions [8,9]. To successfully fight transposons, genome defence systems must be adaptable to recognize many different transposon types. At the same time, these systems must have high fidelity in order to silence only foreign elements and not host genes**.**

How do genomes differentiate their own DNA (self) from foreign transposable DNA (non self)? Data from several organisms implicate that small RNA-based pathways are involved in recognition of foreign genetic elements. The early evidence for this connection has come from experiments performed in plants. In plants it has been observed that the expression of a transgene can result in silencing of the transgene itself, and of its endogenous copy when present [10,11]. A similar phenomenon has since been observed in *Neurospora crassa*, described as ‘quelling’ or transgene-induced gene silencing, and *Caenorhabditis elegans* [12–14]. It has been shown that transgene silencing is mediated by small RNAs and Argonaute family proteins [15,16].

### (a) Fission yeast heterochromatin

In the fission yeast *Schizosaccharomyces japonicus*, transposons cluster at centromeres and telomeres and are silenced by the RNA interference (RNAi) machinery [17]. In *Schizosaccharomyces pombe*, the best characterized fission yeast, the small RNA pathway has been shown to act at the chromatin level and is essential for heterochromatin formation at centromeric repeats [18].

**Figure 1.**
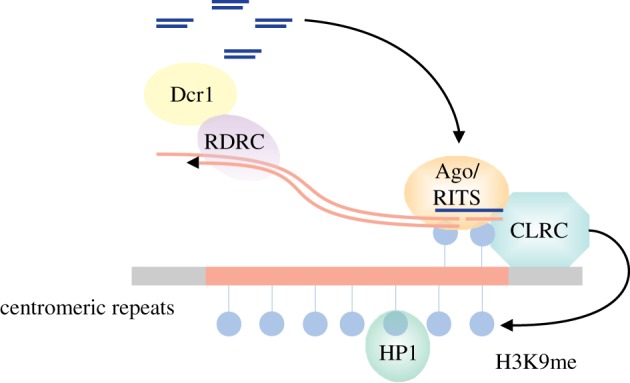
RNAi-mediated transcriptional silencing in *S. pombe*. In *S. pombe*, RNAi induces transcriptional silencing at centromeric repeats that highly resembles transposon silencing in other organisms. Argonaute (Ago) bound small RNAs target the non-coding nascent transcripts and recruit the RNA-dependent polymerase complex (RDRC) and the methyltransferase complex (CLRC). RDRC synthesizes dsRNA which is processed by Dicer (Dcr1) into small RNAs to amplify the signal. Concomitantly, CLRC deposits the heterochromatic silencing mark (H3K9me), which reinforces small interfering RNA (siRNA) generation and establishes heterochromatic silencing.

In *S. pombe*, small RNAs, the mediator of centromeric silencing, direct the inactivation of RNAs by guiding the Argonaute RITS (RNA-induced transcriptional silencing complex) complex to complementary RNA sequences [19]. The RITS complex recruits the methyltransferase complex CLRC to chromatin, which deposits the repressive histone 3 lysine-9 methylation (H3K9me) mark [19–21]. Once deposited, H3K9 methylation recruits Heterochromatin Protein 1 (HP1) family proteins, which leads to heterochromatin formation (figure 1). In RNAi-mediated heterochromatin formation, centromeric transcripts serve as a template for small interfering RNA (siRNA) generation, Argonaute targeting and recruitment of the methyltransferase complex CLRC [18]. At the same time heterochromatic transcripts are degraded by the RNAi machinery and the Ccr4-Not complex, which is required to maintain heterochromatic silencing [22].

Why does RNAi target only centromeric repeats for heterochromatic silencing in fission yeast and what provides the fidelity? Although on average *S. japonicus* and *S. pombe* have approximately 55% amino acid identity, the centromeric sequences are completely different, but targeted by RNAi in both organisms [17]. This shows that heterochromatic repeats evolve much faster than protein coding genes and that there is no evolutionary pressure to maintain the sequence of these regions. How can such rapidly evolving sequences be recognized and silenced?

### (b) Argonaute surveillance in fission yeast

Small RNA-based mechanisms have been shown to be key players in protecting the genome against repetitive and transposable elements, especially in the germ line. How can small RNA-based pathways discriminate repeats and transposons from the host genes? In *S. pombe*, we have observed that Argonaute binds a class of Dicer-independent small RNAs called primal RNAs (priRNAs) [23,24]. priRNAs are generated from single stranded RNAs and resemble to a large extent the transcriptome of the cell. Although priRNAs are generated from many genomic loci, RNAi is restricted to centromeric repeats. This is due to the high level of sense and antisense transcripts arising from the centromeric repeats that are turned into priRNAs. In order to be functional small RNAs need to be in the antisense orientation to the transcript they target. These antisense priRNAs can base-pair with the sense transcript and guide Argonaute to the RNAs transcribed from centromeric repeats. On the contrary, euchromatic protein coding genes generate mostly sense priRNAs and only very low levels of antisense transcripts and antisense priRNAs which would be required for initiation of silencing [23].

In this model, priRNAs guide Argonaute to the centromeric repeats where it recruits the H3K9 methyltransferase complex CLRC [25–27]. CLRC deposits then the initial H3K9 methylation, which is used as nucleation for heterochromatin establishment. In agreement with the model, we have observed that priRNAs are capable of guiding Argonaute to centromeric repeats and of inducing low levels of H3K9 methylation [23]. Concomitantly, Argonaute recruits the RNA-dependent polymerase complex RDRC [28], which synthesizes dsRNA which is processed by Dicer into secondary siRNAs. This will amplify the signal and lead to heterochromatin formation (figure 1).

**Figure 2.**
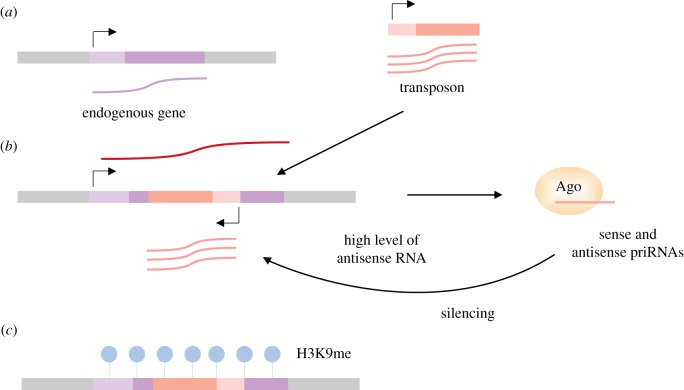
Transposon insertions perturb transcription which could be detected by Argonaute surveillance mechanism. Transposon silencing is essential for genome stability and cell growth. Cells have evolved different strategies to efficiently accomplish this task. How transposons are initially recognized and targeted remains to be determined. We propose that the small RNA pathway can detect perturbation of the transcription patterns. The random insertions of a transposon into the host genome can generate a high level of antisense transcripts. For example this can be caused by the transposon insertion in the opposite orientation to an endogenous transcript (*a*,*b*). This would result in the generation of both sense and antisense RNAs (*b*). In our model these transcripts would then enter in the priRNA pathway and guide Argonaute to the locus of the transposon insertion (*b*) to induce transcriptional silencing (*c*).

In the proposed model Argonaute scans the transcriptome degradation products and initiates silencing at places with a high level of antisense transcripts. It is tempting to speculate that the priRNA-based surveillance mechanism could detect insertion of a new active transposon. Because of the transposition to different genomic locations, it is likely that new transposons will generate high levels of antisense transcripts. For example, insertion of a transposon in a reverse orientation into or near another transcript will generate sense transposon transcripts and antisense transcripts from the existing genomic promoter. This will lead to generation of antisense priRNAs which might accumulate to a sufficient level to initiate silencing of the element. The Argonaute transcriptome surveillance may play an essential role in genome defence and initial recognition of transposable and other invading genomic elements (figure 2).

These observations suggest that increased levels of antisense transcripts might lead to ectopic RNAi. Previously it has been reported that the nuclear exosome subunit Rrp6 is involved in degradation of many antisense transcripts [29]. To test the possibility that antisense transcripts might initiate RNAi, we have perturbed the system by deleting the Rrp6 nuclease. In *rrp6* deletion cells we have observed ectopic siRNA generation and heterochromatin formation at protein coding genes and non-coding RNAs [24]. These results show that exosome-mediated RNA quality control protects the genome from spurious RNAi.

This is consistent with the hypothesis that high amounts of antisense RNAs might be the trigger for transposon recognition and silencing. To protect protein coding genes from RNAi, cells have evolved elaborate RNA quality control mechanisms that rapidly remove antisense transcripts. In the absence of these quality control pathways, RNAi can be recruited and induce silencing at the wrong genes. Our data suggest that Argonaute associates with random degradation products to generate priRNAs, which scan the transcriptome and can nucleate RNAi and heterochromatin in a process of genome defence.

### (c) Argonaute surveillance in *Caenorhabditis elegans*

In *C. elegans*, Piwi-interacting RNAs (piRNAs) have been suggested to scan for foreign DNA and subsequently nucleate siRNA generation and heterochromatin formation. Primary piRNA, also called 21U RNAs, are transcribed by RNA polymerase II as short transcripts and bind PRG-1, a *C. elegans*-specific Piwi protein. *Caenorhabditis elegans* piRNAs lack obvious targets and have the potential to silence a wide plethora of transcripts. Despite the abundance of possible targets, *C. elegans* piRNAs are mainly targeting transposons and transgenes [30–35]. Recent studies show that *C. elegans* piRNAs can interact with all germline mRNAs, but the endogenous transcripts are protected from silencing. The worm-specific Argonaute CSR-1 recognizes endogenous mRNAs and acts upstream of PRG-1, preventing its binding to expressed genes. Moreover, specific sequences found in introns and promoters confer resistance to piRNA silencing [36,37].

Similar to fission yeast priRNAs, *C. elegans* piRNAs guide PRG-1 to complementary targets and recruit the RNA-dependent RNA polymerase RdRP to produce secondary 22G RNAs. 22G RNAs are loaded on worm-specific Piwi protein (Wago-1) and mediate silencing in an analogues way to siRNAs in fission yeast [32,33,38]. Small RNAs guide the Argonaute family of proteins to chromatin and recruit histone modifying enzymes that deposit H3K9 methylation and mediate transcriptional silencing [35]. The piRNA/22G RNA pathway in *C. elegans* highly resembles the priRNA/siRNA pathway in *S. pombe*.

### (d) piRNA system of *Drosophila*

An analogous system acts in *Drosophila* where Dicer-independent piRNAs protect the germline genome from mobile elements [39,40]. In the absence of the piRNA pathway, transposon RNA levels increase in *Drosophila melanogaster* germline cells, leading to sterility. piRNAs, like priRNAs in fission yeast, are a unique class of small RNAs generated in a Dicer-independent way. These small RNAs originate from single stranded RNAs transcribed by the RNA polymerase II from piRNA clusters, which consist of defective transposons, rather than double stranded RNAs [41–43]. The long RNAs transcribed from these piRNA clusters are processed into primary piRNAs [44]. Primary piRNAs guide the Piwi family of proteins, a clade of Argonaute proteins, to transposons and initiate the generation of secondary piRNAs in order to amplify the signal [39]. Like in fission yeast, in the nucleus piRNAs guide Piwi to chromatin where it recruits chromatin modifying enzymes that deposit H3K9 methylation and establish transcriptional silencing [45–47]. The piRNA system successfully defends germline cells from existing transposon; however, how this system can recognize new transposons remains to be determined.

Through evolution, fragments of transposons have been placed into piRNA clusters and have served as a memory for silencing. The piRNA clusters are mainly localized at subtelomeric or pericentromeric heterochromatin and code for the majority of piRNAs [39,40]. How were transposons initially recognized and subsequently placed into piRNA clusters? One possibility is that after recognition, transposons are actively moved into piRNA clusters to maintain the memory of silencing. Another possibility is that transposons eventually insert themselves into the clusters by random transposition. Experiments done in flies and mice show that the insertion of an ectopic sequence into a piRNA cluster results in the production of piRNAs [48]. This resembles the fission yeast silencing system, where insertion of an ectopic sequence into pericentromeric heterochromatin leads to siRNA generation and silencing [23,49].

In another study the authors have taken advantage of the *Drosophila* hybrid dysgenesis phenotype, for which sterile progeny arise when crossing a naive female with a male encoding for different transposons [50]. The introduction of the paternal P element transposon into a naive female strain leads to transposon mobilization and reduced fertility. As the hybrid females age the fertility is restored and transposons are silenced by piRNAs produced from the paternally inherited piRNA clusters. It is important to mention that new transposon insertions into the clusters have been observed as well [50]. These studies show that the insertion of a DNA sequence into small RNA generating clusters will initiate silencing of this element. It remains to be determined how piRNA clusters in *Drosophila* or siRNA generating sequences in *S. pombe* are defined. When the organism encounters a new transposon, how is this element recognized and eventually inserted into a silent cluster?

### (e) Transposon defence in plants

In plants RNAi pathways are involved in regulation of genome expression and constitute the primary defence mechanism against transposons and viruses. In *Arabidopsis thaliana* the RNA directed DNA methylation pathway (RdDM) establishes cytosine DNA methylation at transposable elements [51,52]. Existing transposons are transcribed by the specialized RNA polymerase IV, which recruits the RNA-dependent RNA polymerase 2 to generate dsRNA and 24 nt long siRNAs [53,54]. siRNAs are then loaded onto Argonaute proteins, guide the silencing machinery to the nascent transcripts and direct DNA methylation and heterochromatin formation to silence transposons [55]. The RdDM pathway requires the specialized RNA polymerases IV and V to transcribe transposons, Argonaute proteins and the small RNAs.

How transposon transcripts are initially recognized as aberrant is an intriguing question. A recent work on *A. thaliana* suggests that the first defence against invading elements is mediated by the post-transcriptional silencing pathway. The transposon transcripts are recognized and degraded by 21–22 nt long siRNAs and Argonaute proteins [56]. Once the transposon copy number reaches a threshold, the silencing mechanism shifts to a more robust transcriptional silencing. The authors suggest that this is due to the accumulation of dsRNAs, which exceeds the processing capacities of Dicers DCL2 and 4, which act in the post-transcriptional pathway. These dsRNAs are then accessible to Dicer DCL3, which produces 24 nt long siRNAs and feeds the transcriptional silencing pathway, which establishes DNA methylation and heterochromatin.

During reprogramming of the germ line, existing transposons are reactivated and can be targeted by many microRNAs (miRNAs). These miRNAs recruit the RNA-dependent RNA polymerase to initiate generation of 21 nt long siRNAs and transposon silencing [57]. These data suggest that miRNAs can act as a backup mechanism to target transposons for silencing.

A class of small RNAs, called sidRNAs (siRNAs independent of DCLs), is generated independently of Dicer and predominantly map to transposable elements and repeats in plants [58]. sidRNAs are produced by transgenic loci or active transposable elements and can guide Argonaute to the target to direct DNA methylation [58], suggesting that sidRNAs might be the initial trigger for RdDM. In this model, RNA polymerase II transcripts are loaded onto Argonaute and processed into sidRNAs, similar to priRNAs in fission yeast [24]. Like priRNAs, sidRNAs can induce H3K9 and DNA methylation at the target locus with no requirement for prior modifications. This initial H3K9 and DNA methylation can subsequently recruit RNA polymerase IV and RNA-dependent RNA polymerase 2 to produce secondary siRNAs and reinforce the silencing [59].

### (f) Other RNAi-based strategies for transposon recognition

**Figure 3.**
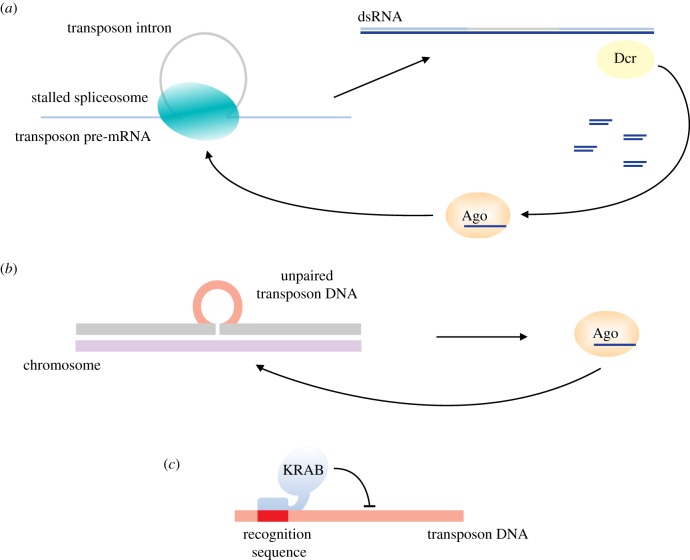
Strategies to detect transposon insertions. In *Cryptococcus neoformans* the stalled spliceosome on transposon transcripts recruits the RNAi machinery to direct silencing (*a*). In *Neurospora crassa* RNAi targets DNA sequences, which are unpaired during meiosis (*b*). In human cells protein-based strategies have been suggested to play a role in recognition of new transposons. In particular, rapidly evolving KRAB zinc-finger proteins might be able to detect new transposons and target them for silencing (*c*).

Another possibility to distinguish host genes from invaders might come from differences in processing between host and transposon RNAs. The RNA of invading transposons might not be optimized for processing in the host cell, which might be used to distinguish transposons from host genes. In *Cryptococcus neoformans*, another yeast, it has been observed that transposon introns have different splicing kinetics compared with the host introns. This leads to stalling of the spliceosome and recruitment of the RNAi machinery to transposons (figure 3*a*). This suggests that the stalled spliceosomes, or in general, difference in efficiency in RNA processing, could be used by the host to distinguish self from non self [60].

Another interesting strategy to determine newly inserted transposons is the recognition of unpaired DNA sequences during meiosis. For example, it has been shown that a DNA sequence that is unpaired during meiosis is silenced in an RNAi dependent way in *N. crassa* (figure 3*b*) [61]. This meiotic silencing is also able to recognize new transposons [62]. In a similar way a multigenerational small RNA-induced epigenetic silencing (RNAe) in *C. elegans* can recognize and silence a transgene which is in a hemizygous state during meiosis for several generations [63].

### (g) Protein-based transposon defence

In addition to RNAi-based silencing mechanisms, other pathways have been described to be involved in transposon silencing. In *S. pombe* transposons are silenced by the homologue of human CENP-B DNA binding protein, which evolved from a DNA transposase [64]. It is possible that transposons were initially recognized by RNA-mediated silencing pathways, and DNA binding proteins have evolved to assure more robust silencing. In this perspective, evolution of DNA binding proteins would be a second step in transposon repression. In the absence of RNA degradation by the exosome, the Tf2 element in *S. pombe* is indeed targeted by RNAi, which might be reminiscent of its initial recognition [24,65]. These data show that fission yeast cells evolved DNA binding proteins that target the Tf2 RNA to exosome degradation, before it becomes an RNAi target. The evolution of DNA binding proteins might have allowed a more efficient and cost-effective silencing, and reduced transposon proliferation in *S. pombe.* As compared with *S. japonicus*, *S. pombe* has only two transposable elements, silenced by CENP-B homologous proteins, while *S. japonicus* has 10 families of gypsy-type retrotransposons silenced by the RNAi machinery [17].

In human cells, rapidly evolving KRAB zinc-finger proteins have been suggested to be involved in recognition of new transposable elements [66]. The rapid evolution of these proteins might take over some of the functions of RNA-based pathways (figure 3*c*).

## 2. Biogenesis of Dicer-independent small RNAs

Current data indicate that Dicer-independent small RNAs are involved in recognition of transposable and repetitive elements in many organisms. How are these single stranded small RNAs generated? The fission yeast data [23] suggest that priRNAs are random degradation products of the transcriptome, indicating that their precursors are of variable length. This suggested that a 3′ end trimming step is necessary to determine the final length of priRNAs and other single stranded small RNAs.

In fission yeast, Argonaute is required for priRNA generation, but its slicer activity is dispensable [23]. This suggested that Dicer-independent priRNAs are loaded on Argonaute as longer precursors that are trimmed to the final length by a nuclease. More recently, we have identified the 3′–5′ exonuclease Triman (*tri1*), which trims priRNAs and siRNAs to the mature length [24]. Triman belongs to the PARN family of ribonucleases, which are conserved in higher eukaryotes. Recently, PARN-like nucleases have been shown to process piRNA in *C. elegans*, in silkworms and in mammalian cells [67–69]. The biogenesis of Dicer-independent small RNAs in *S. pombe* resembles the biogenesis of piRNAs in animals, indicating high similarity between these pathways.

**Figure 4.**
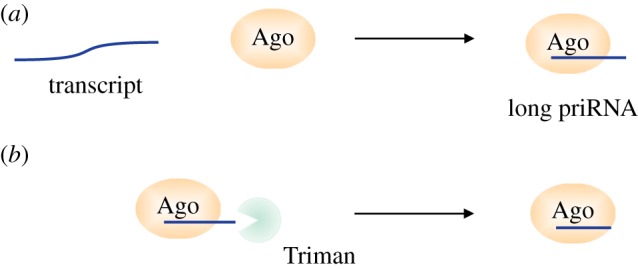
Biogenesis of Dicer-independent small RNAs in *S. pombe* resembles the biogenesis of piRNAs in animals. In *S. pombe* single stranded transcripts of variable length are bound by Argonaute in a process of transcriptome surveillance (*a*). In order to be functional the long Argonaute bound priRNAs need to be trimmed to the mature length. This is achieved through the cooperative activity of Argonaute and the trimming enzyme, Triman, in fission yeast (*b*). In animals and plants small RNA maturation highly resembles the *S. pombe* priRNA processing. In animals, longer single stranded piRNA precursors are bound by a member of the Argonaute family of proteins and processed to the final length by PARN or other nucleases. In plants, Argonaute 4 bound small RNAs are trimmed by the exonucleases Atrimmer1 and Atrimmer2 (*b*).

How does the 3′–5′ exonuclease generate small RNAs of a defined size? Our data show that the priRNA length is determined by the cooperative activity of Argonaute and Triman. Argonaute binds longer priRNA precursors and recruits Triman to process them to the final length, which is defined by the interaction of the priRNA 3′ end with Argonaute (figure 4) [24]. Why do the longer precursors need to be trimmed, sometimes by only few nucleotides? First, we have observed that RNAs longer than 28 nt bind Argonaute with low affinity and eventually dissociate. In this case the 3′ end of the small RNA is likely too far from the Argonaute PAZ domain and does not bind Argonaute [24]. RNA precursors that are 24–28 nt long are stably bound to Argonaute, but are still trimmed to the final length of 22 nt. We have observed that longer small RNAs accumulating in *tri1*Δ cells (24–28 nt) are less functional in guiding Argonaute to slice the complementary targets. This suggests that longer small RNAs interact with Argonaute in a different mode, which does not allow proper positioning of the target RNA in the active site. Likely, the interaction of the 3′ end of a slightly longer small RNA (24–28 nt) with the PAZ domain positions the RNA away from the catalytic site and prevents the cleavage. Our data show that 3′ end trimming of small RNAs to their final length is required for Argonaute slicer activity.

We have shown that priRNAs could re-establish a very low level of H3K9 methylation at centromeric *dg* repeats [23,24]. This low level of H3K9 methylation might serve as a nucleation point for initiation of siRNA generation and establishment of centromeric heterochromatin. This implies that in the absence of priRNAs, heterochromatin establishment might be impaired. In agreement with this hypothesis, we have observed that Triman activity is required for heterochromatin establishment at centromeres, at the *mat* locus and for the maintenance of facultative heterochromatin [24].

Recently, it has been shown that the Triman homologue PARN-1 trims 21U small RNAs in *C. elegans* [69]. Similarly to *S. pombe*, longer 21U precursors are loaded on PRG-1 and are trimmed to the final length. The authors have also observed that longer 21U small RNAs show reduced activity and are less effective in inducing generation of secondary 22G small RNAs [69]. The PARN nuclease PNDCL1 has been shown to trim small RNAs in the silkworm *Bombyx mori* [68]. PNDCL1 has an additional putative transmembrane domain which mediates its mitochondrial localization. Similar to *S. pombe*, 3′ end trimming is important for Piwi slicer activity and longer piRNAs are not methylated and are less stable [68]. PARN family nucleases have been recently shown to trim piRNAs in mouse germ-line cells [67], indicating that this pathway is conserved in most eukaryotes.

Some organisms have lost the PARN-mediated small RNA trimming and have evolved species-specific pathways. In *D. melanogaster* the 3′ end of piRNAs is processed by the endonuclease Zucchini and the 3′ exonuclease Nibbler [70–73]. Although the nucleases are different, the mechanism resembles the small RNA processing in *S. pombe.* In *A. thaliana* AGO4 bound small RNAs are trimmed to the mature length by the 3′–5′ exonucleases Atrimmer1 and Atrimmer2 [58]. Loss of Atrimmers leads to reduced sidRNA production, suggesting that trimming stabilizes sidRNA precursors which otherwise would dissociate from AGO4 and would be degraded.

The data show that in many organisms longer single stranded precursors are bound by a member of the Argonaute family of proteins and are processed to the final length by PARN or other nucleases.

## 3. 3′ end tailing and degradation of small RNAs

Small RNA-based silencing pathways need to discriminate between self and non self and should induce silencing exclusively at transposable and repetitive elements. It is essential for cell survival that RNAi is directed only to its proper targets and not to protein coding genes. The fidelity of small RNA silencing pathways is achieved by various RNA quality control mechanisms that degrade aberrant transcripts. This is fundamental to assuring the presence of only fully functional species and degrading the noise small RNAs that might arise from other loci.

We and others have observed that non-templated nucleotides are added to the 3′ end of small RNAs (RNA tailing) [23,74–77]. Several studies have highlighted the connection between 3′ tailing and small RNA quality control. The addition of non-templated nucleotides affects small RNA stability and modulates their activity. Small RNA tailing can be developmental or tissue specific and the same modification can have different effects depending on the RNA target [78]. Small RNA degradation can be initiated at the level of small RNA precursors, mature small RNAs and also Argonaute bound small RNAs.

The 3′ end tailing of siRNAs was first observed in *A. thaliana*, where the nucleotidyl transferase HESO1 adds non-templated uridines to the 3′ end of miRNAs and siRNAs and targets them for degradation [79–82]. In plants uridylation of siRNAs and miRNAs promotes their degradation [82], while miRNA adenylation has been suggested to have the opposite effect [75]. More recently it has been shown that HESO1 and URT1 can tail Argonaute bound miRNAs [83,84] and even though the tailed miRNA remains associated with Argonaute, its slicing activity is reduced. These data show that tailing of small RNAs modulates their activity, which is consistent with our observation that longer small RNAs show poor slicer activity in fission yeast [24]. In many organisms small RNAs can be protected from tailing and degradation by 2′-*O*-methylation at the 3′ end mediated by the methyltransferase HEN1. Recent data indicate that 2′-*O*-methylated miRNAs in plants are first trimmed at the 3′ end by SDN1, and then tailed by HESO1 [85].

In worms and mammals, the RNA binding protein Lin28 recruits the nucleotidyl transferases TUT4 and TUT7 to the pre-*let-7* miRNA, leading to pre-miRNA oligouridylation and degradation by the exonuclease Dis3L2 [76,79,86,87]. In *C. elegans* the nucleotidyltransferase CDE-1 is responsible for the uridylation of siRNAs that are loaded onto CSR-1 [88]. The authors suggest that small RNA uridylation limits the loading of RdRP EGO-1 generated siRNAs into CSR-1. This restricts EGO-1 generated small RNAs to the chromatin associated small RNA pathway and separates them from endogenous RNAi.

In mammalian cells mono-uridylation of some miRNAs has been shown to facilitate their biogenesis by creating a 2 nt overhang, which is optimal for Dicer activity [89]. On the contrary, defective Argonaute bound pre-miRNAs are oligo-uridylated to facilitate their degradation by the exosome [90]. Uridylation and degradation of Argonaute bound pre-miRNA provides a miRNA loading quality control and prevents Argonaute clogging with defective species. In the alga *Chlamydomonas reinhardtii* the terminal nucleotidyltransferase MUT68 tails siRNAs and miRNAs that lack 2′-*O*-methylation at the 3′ end [91]. This suggests that MUT68 uridylation is involved in quality control and turnover of defective small RNAs.

**Figure 5.**
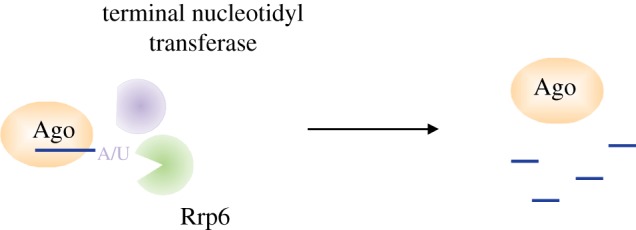
3′ end tailing plays a fundamental role in small RNA quality control and turnover. Argonaute bound small RNAs are tailed at the 3′ end by terminal nucleotidyl transferases. In *S. pombe* Argonaute bound small RNAs uridylated or adenylated by the terminal nucleotidyl transferases Cid14 and Cid16 are actively removed from Argonaute and degraded by Rrp6. This small RNA turnover is necessary to protect the cells from uncontrolled RNAi.

RNA quality control pathways have been implicated in RNAi-mediated heterochromatin formation in fission yeast. It has been suggested that the TRAMP complex targets abundant RNAs to the exosome to prevent their entering into the RNAi pathway [92]. Later it was shown that in the absence of the nuclear exosome, RNAi targets mRNA transcripts that are normally not targeted [24,65]. Our recent work has shown that in *S. pombe* Argonaute bound small RNAs are tailed at the 3′ end. This 3′ end tailing of small RNAs leads to their dissociation from Argonaute and degradation by the nuclear exosome, adding another layer in quality control [93]. We have shown that the non-canonical poly(A)-polymerase Cid14 adds non-templated adenines, while the uridyl-transferase Cid16 adds non-templated uridines to Argonaute bound small RNAs. Cid14 is a well characterized member of the TRAMP complex. This suggested that Cid14 might recruit the exosome to degrade Argonaute bound small RNAs. We have shown *in vitro* that Cid14 and Cid16 activities recruit the nuclear exosome Rrp6 and mediate the degradation of Argonaute bound small RNAs (figure 5) [93]. In agreement with the *in vitro* data, we have observed *in vivo* that in *cid14* deletion cells Argonaute bound siRNAs have a longer half-life compared with wild-type cells. These data show that Cid14 and Cid16 tail Argonaute bound small RNAs, and actively remove them by recruiting the nuclear exosome. This small RNA turnover will mainly affect the least abundant small RNAs, keeping them below the threshold required to initiate silencing. On the contrary, centromeric siRNAs are constantly produced and this compensates for their degradation.

## 4. Loss of fidelity in the RNAi pathway

What happens to the cells when quality control mechanisms are missing, small RNAs are not actively removed from Argonaute and noise small RNAs accumulate? We have observed that in *cid14*, *cid14cid16* and *rrp6* deletion cells, Argonaute is associated with a higher amount of small RNAs [24,93]. Moreover, new classes of small RNAs appear, such as priRNAs arising from many mRNAs, introns and non-coding RNAs. At some loci these priRNAs initiate the generation of secondary siRNAs and ectopic silencing of euchromatic genes. Although these small RNAs can silence their target genes, they do not establish heterochromatin, with the exception of the ribosomal DNA locus where we observed an increase in H3K9 methylation in *cid14* deletion cells. This suggests that siRNAs at ectopic loci target RNAs post-transcriptionally and that the chromatin remains refractory to H3K9 methylation and heterochromatin formation in *cid14*Δ and *cid14*Δ*cid16*Δ cells. Our data show that small RNA turnover is necessary to reduce noise in Argonaute bound small RNAs. This prevents Argonaute targeting to ectopic loci and protects the cells from uncontrolled RNAi.

Uncontrolled RNAi that targets protein coding genes required for normal cellular functions is clearly not advantageous for the cell growth. We have observed that in *cid14*Δ, *cid14*Δ*cid16*Δ and in *rrp6*Δ cells, RNAi targets *rdp1*. Rdp1 is the *S. pombe* RNA-dependent RNA polymerase required for dsRNA synthesis and siRNA generation [23,28,94]. It is particularly interesting that in these cells RNAi targets a gene essential for RNAi itself. One possibility is that *rdp1* silencing reduces the efficiency of the RNAi machinery and protects the genome from an even more deleterious uncontrolled RNAi. Consistent with this, Rdp1 over-expression in *cid14*Δ cells strongly reduces their viability, indicating that the reprogramming of *rdp1* expression is essential for cell viability [93]. It is likely that in these cells *rdp1* was randomly targeted by RNAi, and only the fittest cells that silenced *rdp1* were selected. It seems that silencing of *rdp1* can provide a balance between functional centromeric RNAi and restricted ectopic RNAi. These data show that yeast cells can use RNAi to reprogramme their genome expression to adapt to external or internal stresses. In cancer cells, epigenetic variations might enable tumour cells to adapt to stress conditions and to survive therapies [95,96].

## 5. Conclusion

The balance between the host genome and transposable elements is delicate and poses several problems to the host. On one hand the host needs to silence these elements in order to prevent their mobilization. On the other hand, transposons are important drivers of genome evolution, which could be beneficial to the host, especially in stress conditions. The data show that many organisms adopted small RNA-based strategies to solve this problem. Even though there are differences between organisms, the basic strategy seems to be conserved. We propose that Argonaute proteins scan the transcriptome and recruit the silencing machinery to regions with irregular transcription. In this model, the RNAi machinery can sense perturbation of the transcriptome caused by the mobilization of transposable elements. At the same time scanning primal small RNAs need to be constantly degraded to provide fidelity. RNA quality control mechanisms prevent accumulation of background primal small RNAs and protect the genome from detrimental ectopic RNAi. These data show that small RNA-based silencing pathways are extremely plastic and versatile and, because of this, need to be tightly regulated.

## Data accessibility

This article has no additional data.
